# Microbial Pattern Recognition Causes Distinct Functional Micro-RNA Signatures in Primary Human Monocytes

**DOI:** 10.1371/journal.pone.0031151

**Published:** 2012-02-17

**Authors:** Robert Häsler, Gunnar Jacobs, Andreas Till, Nils Grabe, Christian Cordes, Susanna Nikolaus, Kaiqin Lao, Stefan Schreiber, Philip Rosenstiel

**Affiliations:** 1 Institute of Clinical Molecular Biology, Christian Albrechts University Kiel, Kiel, Schleswig-Holstein, Germany; 2 Department of Otorhinolaryngology, Head and Neck, University Hospital Schleswig-Holstein, Kiel, Schleswig-Holstein, Germany; 3 First Department of Medicine, University Hospital Schleswig-Holstein, Kiel, Schleswig-Holstein, Germany; 4 Applied Biosystems, part of Life Technology, Foster City, California, United States of America; McGill University, Canada

## Abstract

Micro-RNAs (miRNAs) are short, non-coding RNAs that regulate gene expression post transcriptionally. Several studies have demonstrated the relevance of miRNAs for a wide range of cellular mechanisms, however, the current knowledge on how miRNAs respond to relevant external stimuli, e.g. in disease scenarios is very limited. To generate a descriptive picture of the miRNA network associated to inflammatory responses, we quantified the levels of 330 miRNAs upon stimulation with a panel of pro-inflammatory components such as microbial pattern molecules (flagellin, diacylated lipopeptide lipopolysaccharide, muramyl dipeptide), infection with *Listeria monocytogenes* and TNF-α as pro-inflammatory control in primary human monocytes using real time PCR. As a result, we found distinct miRNA response clusters for each stimulus used. Additionally, we identified potential target genes of three selected miRNAs miR-129-5p, miR-146a and miR-378 which were part of PAMP-specific response clusters by transfecting THP1 monocytes with the corresponding pre- or anti-miRNAs and microfluidic PCR arrays. The miRNAs induced distinct transcriptomal signatures, e.g. overexpression of miRNA129-5p, which was selectively upregulated by the NOD2-elicitor MDP, led to an upregulation of DEFB1, IRAK1, FBXW7 and IKK γ (Nemo). Our findings on highly co-regulated clusters of miRNAs support the hypothesis that miRNAs act in functional groups. This study indicates that miRNAs play an important role in fine-tuning inflammatory mechanisms. Further investigation in the field of miRNA responses will help to understand their effects on gene expression and may close the regulatory gap between mRNA and protein expression in inflammatory diseases.

## Introduction

Originally, when micro-RNAs (miRNAs) were first discovered in *Caenorhabditis elegans* [1], they were thought to be an oddity in gene regulation of nematodes. Further research however, showed that miRNAs are present in a large number of eukaryotes, from plants to humans. Generally, miRNAs consist of 19–24 nucleotides and are highly conserved across species [2]. Currently, more than 5900 miRNAs have been identified and deposited in the miRNA database [3]–[5]. miRNAs can be encoded anywhere in the genome [6], and current data indicates that only 2% of the human genome encode for 30,000 protein-coding mRNAs, whereas 60–70% of our DNA is transcribed into non-coding RNA [7], [8]. Recent genome-wide computational screens for miRNA targets in humans predict that at least 10% [9] to 30% [10] of all genes are regulated by miRNAs. These predictions suggest that a single miRNA can suppress up to hundreds of target mRNAs, while one target mRNA can be controlled by several miRNAs. Consequently, miRNAs are being discussed as a new type of post-transcriptional regulatory mechanism [2].

As a result of their regulatory nature in healthy physiology, miRNAs have significant impact on diseases, such as cancer [11]–[13] and infectious diseases, where pathogens express miRNAs to interact with the host organism [14].

Due to their biochemical properties, miRNAs have the potential to be exploited as novel therapies in a wide range of human diseases [15], e.g. mechanisms targeting obesity [16], cancer [17], [18] or inflammation [19]. To properly adjust such treatments, one of the first requirements is to understand which miRNAs play a key role in a given scenario. Currently, there is very limited knowledge about i) which miRNAs are involved in specific processes and physiological responses, ii) at what time point miRNAs start interacting with the target gene and iii) what target genes are influenced by miRNAs in a given scenario, such as disease. In this context, a growing number of studies started to focus on the relevance of miRNAs in inflammation. Stimulation of myelomonocytic cell line THP-1 with lipopolysaccharide (LPS) resulted in upregulation of miR-132, miR-155 and miR-146 [19], [20], which has also been shown to play a potential role in psoriasis [21] or cancer [22].

Following the current hypothesis of miRNAs being a new major regulator of gene expression, and therefore exhibiting a functional impact on many physiological processes, we aimed to investigate the relevance of miRNA signatures in responses to microbial pattern molecules. In a first step, 330 known human miRNAs were quantified in primary human monocytes using TaqMan® real time PCR after stimulation with 5 different stimuli (4 defined pathogen associated molecular pattern molecules (PAMPs): FSL-1, flagellin, LPS, muramyl dipeptide and infection with the intracellular bacterium *L. monocytogenes* at a MOI of 100/cell) at 3 different time points. Selected miRNAs, which were regulated in response to the stimuli, were further analyzed by transfecting THP-1 cells with the corresponding pre- or anti-miRNA, where the effects of the transfection on target-gene level were examined by quantification a set of well-defined inflammation associated transcripts. This is the first attempt to monitor miRNA responses in primary human immune cells after stimulation of innate immune pathways, suggesting that miRNAs act in functional groups and exhibit downstream effects on inflammation-relevant pathways such as TNF-α and NF-κB signaling.

## Materials and Methods

### Extraction and stimulation of monocytes

All individuals included (n = 4, male, median age: 30; age range 26–33) in the study were free of medication, non-smokers and did not consume alcohol within the last 24 h. Blood was sampled at the same daytime to correct for circadian effects. All blood donors agreed to participate by giving written informed consent at least 24 hours before the study. Primary human monocytes were isolated as described before [23]. Viability of monocytes was >95% as determined by trypan blue exclusion and purity was at least 85% as assessed by May-Grünwald/Giemsa staining of cytospins (Merck, Darmstadt, Germany). Procedures related to human material were approved by the ethics committee of the Christian-Albrechts University, Kiel (B231/98). Cells were stimulated with either flagellin (500 ng/ml), diacylated lipopeptide (FSL, 100 ng/ml), LPS (100 ng/ml), living *Listeria monocytogenes* (MOI = 100), muramyl-dipeptide (MDP) (10 µg/ml) or TNF-α (10 ng/ml) for 0, 1, 2, or 4 hours.

Synthetic pre-miR-129-5p, pre-miR-ctrl, anti-miR-146a, anti-miR-378 and anti-miR-ctrl were purchased from Ambion (AppliedBiosystems). Flagellin and FSL (FSL-1) were obtained from InvivoGen (LaJolla, CA, USA), MDP was from Bachem (Heidelberg, Germany), and human TNF-α was purchased from R&D Systems Inc. (Minneapolis, MN, USA). Lipopolysaccharide (LPS) was kindly provided by Ulrich Zähringer (Research Center Borstel, Germany).

The *Listeria monocytogenes* serotype 1/2a strain EGD was used as a model organism for bacterial infection. Primary human monocytes were infected with *Listeria* at a multiplicity of infection (MOI) of 100 bacteria/cell as described before [24].

### Transfection of monocytic cell lines

Human acute monocytic cell line THP-1 (ACC16) was purchased from the German Collection of Microorganisms and Cell Cultures (DSMZ, Braunschweig, Germany). THP-1 cells were cultured in RPMI-1640 (PAA Laboratories, Paschberg, Austria). Medium was supplemented with 10% fetal calf serum (FCS) and penicillin/streptomycin (each at 50 µg/ml), and cells were grown in 5% CO_2_ at 37°C. Transfection of THP-1 cells with miRNA was performed using the Amaxa electroporation system (Lonza/Amaxa, Walkersville, MD, USA) according to the manufacturer's manual. In short, 1×10^6^ cells were transfected with specific miRNA or appropriate control miRNA (5 µM) using Amaxa kit V and electroporation program T-08. Cells were kept at 37°C overnight before stimulation with MDP (10 µg/ml) or TNF-α (10 ng/ml) for 0, 1, 2, or 4 hours.

### Quantification of miRNA responses in primary monocytes

In an initial screen, we quantified 330 individual miRNAs in primary human monocytes, stimulated with 6 different agents (unstimulated control, flagellin, FSL, LPS, *Listeria monocytogenes*, MDP, TNF-α) at 3 different time points (0 h control, 1 h, 2 h and 4 h). Each experiment was repeated with monocytes from all 4 different blood donors.

Micro-RNAs in primary human monocytes were quantified using 384-well format real time TaqMan® PCR (Applied Biosystems) as previously described [25]. Briefly, a monocyte sample representing 100 cells was denatured at 70°C for 1 min, followed by a reverse transcription reaction containing individual stem-loop primers for 330 miRNAs. A pre-amplification was performed which provided the final template for the quantification via TaqMan®. Each sample was measured in duplicates.

### Data analysis

Acquired real time PCR data was analyzed using the 2^−ΔΔC*t*^ method [26]. The experiments were normalized using a scaling method (MedianIQR, software: Spotfire DSMA 9.1, TIBCO Somerville, MA, USA) that adjusts location and scale of the data so expression values of all experiments have equal medians and equal interquartile ranges. Fold-changes were calculated based on the ratio between stimulated sample and unstimulated control; p-values were generated using the Mann-Whitney U-test. To correct for multiple testing and to calculate false discovery rates, we used a resampling based method (Westfall and Young permutation with k = 10 000 permutations) for all observed fold-changes and all observed p-values [27]. Micro-RNAs were considered as significantly responding to a stimulus when they met three criteria: i) present in at least 50% of one experimental group, ii) adjusted p-value ≤0.05 and iii) fold-change <−1.5 or >1.5.

### Similarities of miRNA responses

Expression profiles of all 133 miRNAs that where categorized as present were correlated to each other using the Spearman rank correlation resulting in 8,778 correlation coefficients [28]. In a second step, the correlation coefficients were ordered using hierarchical clustering (clustering method: UPGMA (unweighted average), distance measure: correlation, software: Spotfire DSMA 9.1) in order to generate a map of miRNAs that respond in a similar fashion to the different stimuli.

### Heatmaps and clustering methods

To generate functional response groups of miRNAs, clusters of significantly responding miRNAs were created for each stimulus individually and displayed as heatmaps (clustering method: UPGMA (unweighted average), distance measure: correlation, software: Spotfire DSMA 9.1). Within group variations were calculated using the inter quartile range, normalized to the median of the group.

### In silico prediction of miRNA targets

To predict potential interaction partners of miRNAs *in silico*, 5 different algorithms were applied as previously described: i) miRanda [9], ii) miRanda miRBase [29], iii) Target Scan [10], iv) PicTar [30] and v) PITA [31].

### Quantification of target gene levels

The mRNA expression levels of potential targets and a housekeeping genes (*ß-Actin*) were analyzed in duplicate and quantified by TaqMan® low-density array real-time PCR (Applied Biosystems). Target genes were selected literature based using 4 criteria: i) associated to immune response in general, ii) associated to response to bacterial and/or pathogen stimuli, iii) associated to innate immunity and iv) transcripts, upstream of NFκB activation and/or MAP kinase activation and their interaction partners. In brief, 35 µl of single-stranded cDNA (equivalent to 35 ng of total cellular RNA) were mixed with 15 µl nuclease-free water and 50 µl TaqMan® Universal PCR Master Mix. The total volume of 100 µl was loaded into each sample port of the microfluidic cards that were subsequently sealed and centrifuged at 256× *g* for 10 min. Each card was analyzed on the ABI 7900 HT platform (Applied Biosystems). The thermal cycling conditions were 2 min at 50°C and 10 min at 95°C followed by 40 cycles of 30 s at 97°C and 1 min at 60°C. Further data analysis was carried out as described above for quantification of miRNA responses.

### Gene Ontology analysis

Gene Ontology analysis was performed as previously published [32], applied to predicted target transcripts of regulated miRNAs. Biological processes associated to the transcripts were retrieved from the Gene Ontology Consortium (www.geneontology.org).

## Results

### miRNA response signatures

**Figure 1:**
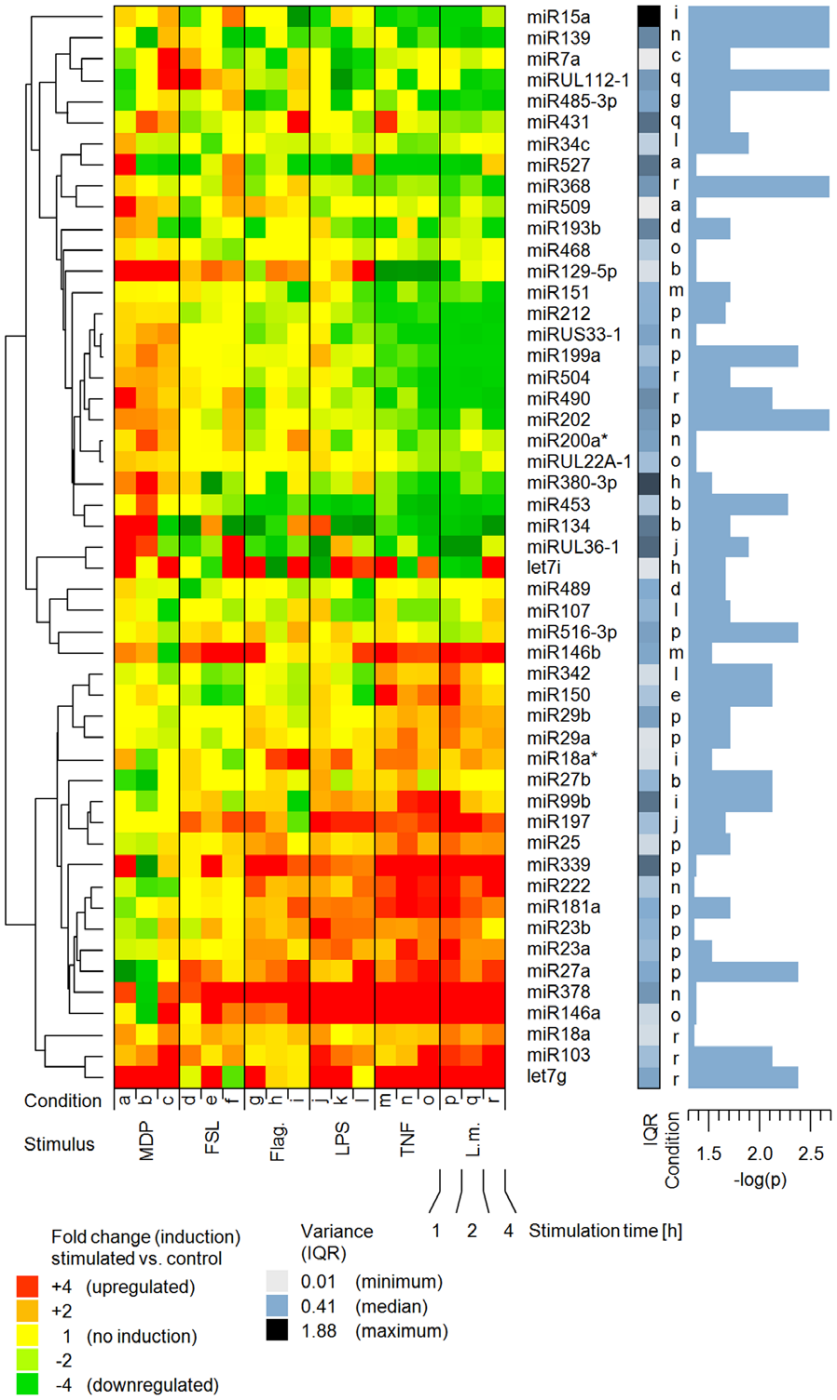
Induction map of miRNA responses in primary monocytes, resulting from 6 different stimuli: flagellin, FSL (diacylated lipopeptide FSL-1), LPS (lipopolysaccharide), L.m. (*Listeria monocytogenes*), MDP (muramyl-dipeptide), TNF-α (tumor necrosis factor alpha), measured at three different timepoints (1 h, 2 h, 4 h). Fold-changes are based on the ratio of the expression values (stimulated sample versus 0 h control). Micro-RNA fold-changes (arranged in rows) for each sample (arranged in columns) are colored according expression induction: red (upregulation), green (downregulation) and yellow (no or weak regulation). Variation between samples are displayed for each miRNA individually by presenting the normalized inter quartile range (IQR; color coded). P-values are displayed as −log(p) (Mann-Whitney u-test, p = 0.05 corresponds to −log(p) = 1.3). IQRs and p-values are displayed for one selected condition which is listed in an additional column (a-r), corresponding to the coding at the bottom row of the heat-map. Micro-RNA names are listed in the right column. The dendrogram (left) shows the similarities between the induction profiles of all significantly regulated miRNAs, based on the correlation.

**Figure 2:**
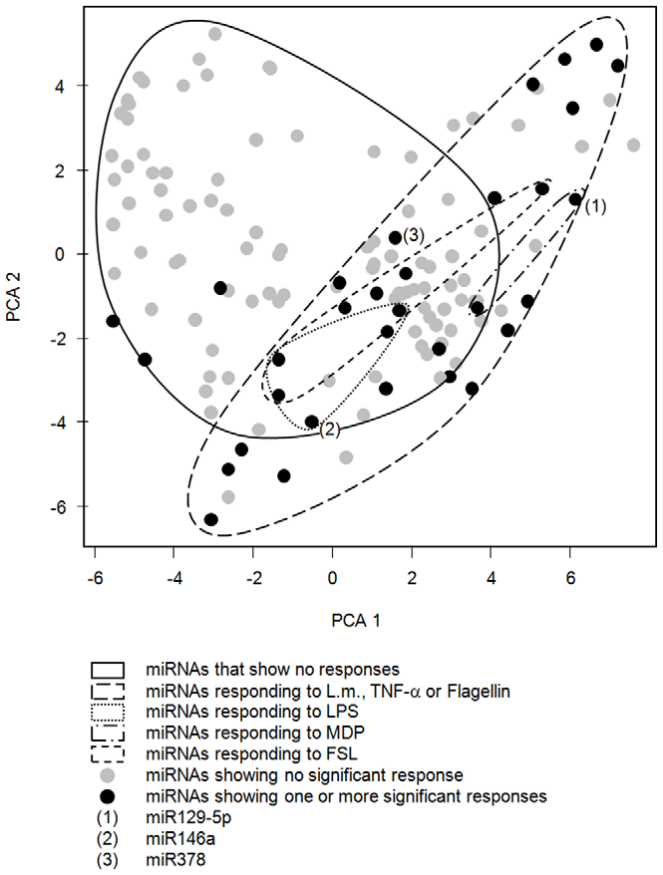
Response patterns of miRNAs and overlap of responses in primary monocytes, based on a principle component analysis on all 133 miRNA categorized as present in the samples, showing the two major components (x and y axis). Each miRNA represents one spot (grey = no significant response; black = one or more significant responses), while groups of miRNA are delimited with lines.

In an initial TaqMan® real-time PCR-based approach, quantification of 330 individual miRNAs showed that 133 miRNAs were present (90^th^ percentile of all measurements <36.5 cycles) in at least 50% of the samples. These miRNAs were subsequently categorized as present and subjected to further analysis. After applying the cutoff criteria (categorized as present and corrected p-value ≤0.05), each of the 6 different stimuli resulted in a specific response signature, consisting of 10 to 35 significantly regulated miRNAs (number of regulated miRNAs: flagellin: 10; FSL: 11; LPS: 15, *Listeria monocytogenes*: 35; MDP: 10 and TNF-α: 27). 61 miRNAs showed a significant regulation in at least one of the conditions examined (Figure 1). A principle component analysis of all 133 present miRNAs (Figure 2) shows similar grouping, displaying unique and overlapping patterns. Moreover, clustering data suggests distinct response patterns reflecting the different nature of the stimuli (Figure S1).

### Similarities of miRNA responses

**Figure 3:**
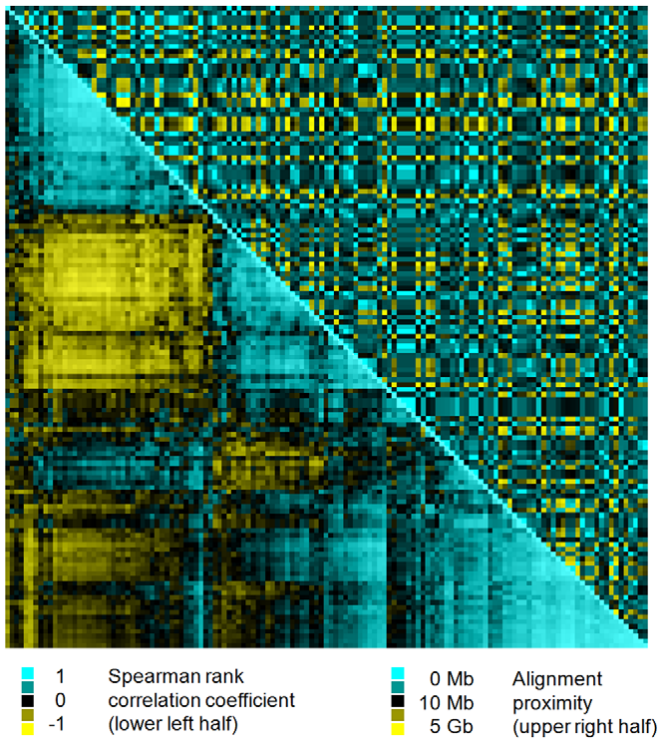
Functional miRNA groups and their representation in genomic clusters. The figure displays, to which extend the similarities in miRNA responses are reflected by their genomic origin. First, 133 miRNAs were selected based on their presence in all samples. Then, these 133 miRNAs were ordered according to their similarities in their responses to pro-inflammatory stimuli (lower half of the cluster; similarity measure: Spearman-rank correlation). Finally, the 133 miRNAs were ordered according to their similarity of their genomic origin (upper half of the cluster; similarity measure: absolute distance in kilobases). When similarities on the response level are mirrored on the genomic level, a group of miRNAs with similar response patterns have a shared genomic origin.

In order to find similarities in the response patterns of individual miRNAs, a correlation analysis were performed which resulted in Spearman rho values from −0.925 to 0.987. For most miRNAs a high correlation of the expression between miRNAs originating from the same stem-loop miRNA could not be observed (Spearman rho range from −0.114 to 0.322). When comparing these correlations to the genomic origin of the miRNAs, which were ordered based on their genomic distance, no corresponding patterns were detected (Figure 3).

### Selection of individual miRNAs

For further analysis, three miRNAs were selected based on their response pattern and based on their significant regulation in response to one or more stimuli (p-value≤0.05). miR-129-5p was the only miRNA that was regulated exclusively in response to MDP (6.02-fold upregulated, p = 0.022) and showed no similarities to response patterns resulting of other stimuli. miR-146a showed a broad response to several stimuli but not to MDP (flagellin: 11.10-fold upregulated, p = 0.0198; LPS 4.01-fold upregulated, p = 0.0198; *Listeria monocytogenes*: 4.64-fold upregulated, p = 0.0077; TNF-α: 5.48-fold upregulated, p = 0.0022). miR-378 showed a pattern similar to miR-146a (*Listeria monocytogenes*: 8.26-fold upregulation, p = 0.0418; TNF-α: 15.69-fold upregulation, p = 0.0297).

### In silico target prediction

The *miRanda* model predicted 3736, 2763 and 2229 targets, *miRBase* predicted 999, 1038 and 1038, *PicTar* predicted 290, 157 and 0, *PITA* predicted 14, 59 and 62 targets and *TargetScanS* predicted 0, 37 and 0 targets for miR-129-5p, miR-146a and miR-378 respectively. A summary of target genes predicted by these 5 algorithms for three exemplary miRNAs (miR-129-5p, miR 146a and miR 378) is presented in Table S2.

### Endogenous miRNA-levels in THP-1 cells

To determine if the transfection of THP-1 cells with the corresponding pre-miRNA or the corresponding anti-miRNA could be expected to lead to functional effects on target-gene level, a quantitative screen of all 330 miRNAs was performed to generate a miRNA-profile in THP-1 cells (Figure S2). Due to its high expression, miR-150 was used as a reference. The profile showed that miR-129-5p was present in low levels (0.02% of miR-150) while miR-146a and miR-378 were expressed at higher levels (both located within the top 15^th^ percentile; Figure S2). Therefore, miR-129-5p was selected to be experimentally increased by transfection of THP-1 cells with pre-miR-129-5p in contrast to miR-146a and miR-378 which were selected to be experimentally decreased by transfection with anti-miR-146a and anti-miR-378, respectively.

### Effects of selected miRNAs on transcript levels of target genes in THP-1 cells

**Figure 4:**
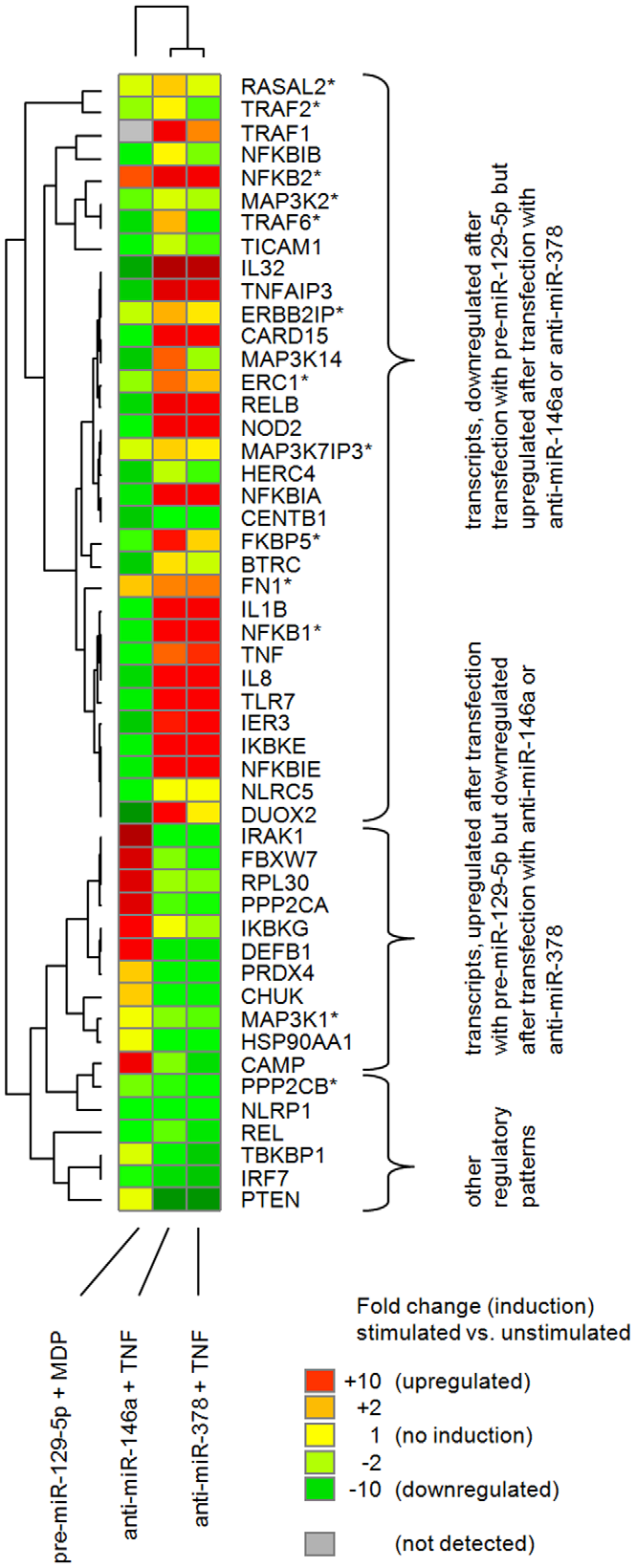
Induction map of potential target genes. Fold-changes of target gene transcripts, as quantified via TaqMan® real time PCR in THP-1 cells, transfected with pre-miR-129-5p and stimulated with MDP, transfected with anti-miR-146a and stimulated with TNF-a and transfected with anti-miR-378 and stimulated with TNF-α are displayed (green = downregulation, yellow = no regulation, red = upregulation, grey = not detected). Target genes are arranged in rows, columns represent the different transfections. The row dendrogram (left) shows the similarities in the expression profiles of the genes, while the column dendrogram (top) shows the similarity between the different stimulations. Gene symbols are displayed right to the heatmap, a star (*) indicates that the observed induction was predicted by at least one algorithm.

From the range of predicted target genes for miR-129-5p, miR-146a and miR-378, a subset of closely interconnected genes, functionally relevant to several inflammatory pathways (e.g. upstream of NFκB activation and/or MAP kinase activation) and interaction partners of these genes were selected for subsequent verification. In total, 132 potential target genes and interaction partners of target genes were analyzed. From those, 51 genes were regulated in response to at least one of the 3 miRNA-transfections. Out of 13 predicted targets for miR-129-5p, 8 showed a downregulation after transfecting THP-1 cells with pre-miR-129-5p (ERBB2IP, ERC1, FKBP5, MAP3K1, MAP3K2, MAP3K7IP3, NFKB1, PPP2CB). THP-1 cells transfected with anti-miR-146a showed an upregulation in 5 of 13 predicted target genes (ERC1, FN1, RASAL2, TRAF2, TRAF6) and transfection with anti-miR-378 resulted in an upregulation of 4 out of 6 predicted targets (ERC1, FKBP5, MAP3K7IP3, NFKB2). These results are presented in Table S1. A cluster analysis of inductions (Figure 4) revealed strong similarities between the two miRNAs that were regulated in response to TNF-α (anti-miR-146a and anti-miR-378; Figure 1), while pre-miR-129-5p exhibited a approximately opposite patter of induced target genes. Quantitative data for selected transcripts after transfection with pre-miR-129-5, anti-miR-146a and anti-miR-378 is presented in Figure S4.

### Biological processes associated to predicted target genes of miRNAs

The target genes, resulting from *in silico* target prediction were summarized into 4 different Gene-Ontology groups and were associated to a different number of biological processes: 14 processes targeted by miRNAs in response to TLR stimuli (represented by LPS, flagellin and FSL; Figure S3A), 13 processes targeted by miRNAs in response to MDP (Figure S3B), 15 processes targeted by miRNAs in response to TNF-α (Figure S3C) and 9 processes targeted by miRNAs showing responses to *Listeria monocytogenes* (Figure S3D). All miRNA groups analyzed resulted in a large proportion (17 of 51) of biological processes associated to intracellular mRNA management.

## Discussion

Fine tuning inflammatory responses, especially in key effector cells like human monocytes, requires regulatory mechanisms which can react to a variety of exogenous and endogenous signals. Current studies indicate that miRNAs play an important role in this context [21], [33], [34], however, the detailed knowledge of how miRNAs act in inflammation is limited to a few prominent candidates. The salient finding of the present study documents that miRNA signatures in response to innate-type microbial pattern recognition in primary human monocytes, which exhibit influence on target genes, may represent an important controlling element. This control may be a crucial element in maintaining and modulating efficient inflammatory responses.

### miRNAs act in groups

Since the exact time-window during which miRNAs will react to a stimulus is not known, the response patterns of 330 miRNAs were quantified at 3 different time points: 1 h, 2 h and 4 h, compared to the 0 h control. The stimuli used (flagellin, FSL, *Listeria monocytogenes*, LPS, MDP and TNF-α) represent a wide range of PAMPs/pro-inflammatory signals relevant to human monocytes. Each of the stimuli resulted in response signatures with a different degree of overlap to other stimuli. The current understanding that one miRNA can control several genes and consequently each gene may be controlled by several miRNAs [35], [36] is in agreement with our observation that larger groups of miRNAs (here 10 to 35) react similar to one stimulus.

### Functional miRNA clusters do not reflect their genomic origin

To test the hypothesis, whether miRNAs signatures represent functional clusters, we performed a correlation analysis of all miRNA signals that were detected in at least 80% of the samples. For each of the stimuli used, a specific response pattern was found (Figure 1 and 2) and the correlation cluster (Figure 3) shows clear and distinct groups. The results support the concept of miRNAs acting in groups [35], [36]. Moreover, closely related miRNAs (e.g. sharing the same stem-loop in their pre-miRNA, like miR-133a and miR-133b) were found to react quite similar to the stimuli applied in our study. However, when inspecting the genomic origin of the miRNAs (Figure 3), we could not find these clusters resulting from operon-like gene structures that are transcribed from a common promoter as previously reported [37], [38]. In this context, it is important to point out, that until now, it has not been experimentally verified whether the previously predicted operonic organization of miRNA loci have a generalized functional significance.

### miRNAs induced by innate immune pathways target specific biological processes

Biological processes associated to the predicted target genes support the current picture of miRNAs as a controlling element in mRNA processing: All miRNA response groups analyzed showed a high proportion of mRNA-processing associated mechanisms. We are aware that Gene Ontology analysis has its limitations due to its literature mining based nature. Similarly, miRNA target gene prediction is a very dynamic field with many open questions. However, both methods are well established and the results indicate their validity as well as the validity of the presented study setup.

### miRNAs that respond to PAMP recognition exhibit potential regulatory effects on inflammation-associated genes under inflammatory conditions

To assess the impact of the presented miRNA patterns, three exemplary miRNAs were selected for further analysis of their impact on potential target transcripts, associated to inflammatory processes: hsa-miR-129-5p, hsa-miR-146a and hsa-miR-378. Several studies reported that miR-146a is associated to the immune response, e.g. being upregulated in response to microbial components and pro-inflammatory cytokines [20], [22] while additional data indicated that miR-146a inhibits TRAF6 and IRAK1 directly [19]. We could support this observation by an *in silico* prediction of targets for miR-146a using 3 different algorithms [9], [29], [30] as well as by experimental approaches *ex vivo* and *in vitro*: i) we observed miR-146a as upregulated in response to stimulation of primary monocytes with TNF-α or LPS (as well as in response to flagellin and infection with *Listeria monocytogenes*) and ii) we observed an upregulation of TRAF6 and IRAK1 in the myelomonocytic THP-1 cell line after blocking miR-146a by transfection of anti-miR-146a in the presence of TNF-α. Generally, *in silico* target prediction approaches should be undertaken with caution, however, this finding documents the validity of the prediction tools as well as our experimental application of anti-miRNAs, when the expected endogenous level of the investigated miRNA is relatively high (e.g. top 20^th^ percentile of the present miRNAs). The observed upregulation of miR-146a in response to several pro-inflammatory stimuli, combined with our findings on inhibitory effects of miR-146a on various inflammation-associated genes could indicate that miR-146a may act as a regulator for tolerance to several pro-inflammatory/PAMP stimuli, which is supported by findings on its role as a negative regulator of innate immune signaling [19], findings on establishment of endotoxin tolerance in monocytes [20] and findings on desensitizing cells to TLR2-dependent activation [39].. Additionally, the concept of miR-146a regulating immune response was supported by a study documenting its potential relevance for adaptive immunity [40].

In this context, it has to be taken into consideration that the presented experimental setup does not allow to exclude differential miRNA responses which are the result of the different stimulatory potential exhibited by the employed stimuli. Consequently, the patterns can be attributed to different stimulatory potential, to specific response mechanisms or to a combination of both.

Based on its signature similarity to miR-146a, miR-378 was selected to be further analyzed on target gene level. Interestingly, miR-378 exhibited this similarity on target-gene level as well: 97 of 117 target genes showed regulation in the same direction (out of 132 genes analyzed, where 15 were not detectable), supporting the hypothesis of miRNAs acting in functional clusters. The observed similarity to miR-146a may also indicate similar functional role in controlling the tolerance to microbial patterns.

As a representative of the MDP-response cluster, miR-129-5p was selected. In contrast to all other miRNAs investigated, miR-129-5p was the only miRNA that was regulated exclusively in response to MDP stimulation and showed no overall similarities to miRNAs in other clusters (as detected by the correlation analysis). Previous reports associate miR-129 mainly with cancer and differentiation processes [41], [42], while its association to inflammation was not described previously. When transfected into monocytes, we observed that the effect of pre-miR-129-5p on transcript levels after MDP-stimulation represents almost the opposite to the effect observed when transfecting cells with anti-miR-146a and anti-miR-378 after TNF-α stimulation (Figure 4). This could illustrate the similar target gene spectrum of TNF-α- and MDP-driven immune responses, since the opposite effects may result from pre- versus anti-miRNA transfection. Moreover, this observation supports the hypothesis of shared mechanisms between TNF-α- and MDP-driven immune responses, which on the other hand are the result of different events. Finally, these findings present miR-129-5p as a novel candidate for NOD-like receptor (NLR)-mediated responses.

The complexity and the potential involvement of interaction partners which were not monitored in this study is demonstrated by the example of nucleotide-binding oligomerization domain containing 2 (CARD15/NOD2): It is downregulated upon transfection of THP-1 cells with pre-miR-129-5p in the presence of MDP, but upregulated upon transfection with anti-miR-146a or anti-miR-378 in the presence of TNF-α. The downregulation however, can not be attributed to miR-129-5p exclusively, since this miRNA responded only to MDP, not to TNF-α. The finding was underscored by findings in THP-1 and HEK293 cells. This suggests either an interaction of several miRNAs or the presence of additional regulatory elements, or both.

In this context, it is unclear to which extent the molecular mechanisms of the three selected miRNAs overlap, however, they exhibit differences on various levels: they show different endogenous levels, *in silico* predictions suggest different target genes and they display different effects on selected target genes in THP cells.

The presented experimental setup does not allow identifying direct interactions between miRNAs and target transcripts for all miRNAs. Experiments like alternating the miRNA binding site on target transcripts will be required to further finemap the regulatory network of miRNAs in health and disease.

It has to be noted, that the observation of changes in the quantity of a target gene transcript can be only observed when the RNA is degraded. In contrast to that, a translational repression, which is the second proposed mode of action for miRNAs, could not be detected with this setup. Since our initial target gene screen detected only differential mRNA expression and not translational repression, it is valid to further follow these results, while keeping in mind that not all effects of the miRNAs could have been monitored.

Moreover, response patterns of miRNAs cannot display a complete picture of the regulatory processes during responses to pro-inflammatory stimuli. Various elements other than miRNAs are involved in this regulation, similarly the selected miRNAs show only a small proportion of potentially relevant regulatory miRNAs. In the same context, assessing the biological impact of miRNAs and their downstream target genes in response to bacterial stimuli requires further studies, ideally conducted in diseased individuals (e.g. sepsis patients). Due to the high inter-individual variation and other limitations of biological material from clinical setting, screening approaches in model systems will remain the method of choice for initial studies.

### Conclusion

Controlling and fine-tuning inflammatory processes is one of the key elements to balance the host organism's response between appropriate defense and excessive immune reaction, which may be harmful. This comprehensive assessment of the miRNome demonstrates that response signatures of miRNAs in primary human monocytes after activation of innate immune pathways may provide a regulatory network to adjust the inflammatory process, while further research may lead to new concepts for therapies modulating innate immunity.

## Supporting Information

Figure S1 **Response clusters illustrating all miRNAs responding significantly to a specific stimulus in primary monocytes.** Flagellin, FSL (diacylated lipopeptide FSL-1), LPS (lipopolysaccharide), L.m. (*Listeria monocytogenes*), MDP (muramyl dipeptide), TNF-α (tumor necrosis factor alpha). Micro-RNA expression (arranged in rows) and for each sample (arranged in columns) are colored according expression intensity: red (high expression), green (low expression). To better visualize the expression differences within one miRNA, colors were based on normalized expression intensity (z-score). Micro-RNA names, signed fold-changes of each gene (+/− representing up/down-regulation) and corrected p-values are listed in the right columns. The dendrogram (left) shows the similarity of the expression profile for each miRNA within its response cluster, based on the correlation.
(TIF)

Figure S2 **Profile of expressed miRNAs in THP-1 cells, illustrating all 330 measured miRNAs and their relative rank position on a 1–100% scale.** To display their relative endogenous expression, miR129-5p, miR-146a and miR-378 are highlighted.
(TIF)

Figure S3 **Biological processes of miRNA target genes.** Results of a Gene Ontology analysis, where predicted target genes of each miRNA were associated to biological processes. The significance of the enrichment or the depletion of a biological process is displayed as −log(p) (orange) and the number of genes observed in the process are displayed on the right (blue, log-scale). Gene Ontology analysis were seperated into 4 different miRNA response groups: Biological processes of target genes of miRNAs regulated by LPS, flagellin and FSL (A), regulated by MPD (B), regulated by TNF-α (C) and regulated by *Listeria monocytogenes* (D).
(TIF)

Figure S4 **Quantitative responses of target genes in THP-1 cells.** Responses were measured after transfecting cells with pre-hsa-miR129-5p, anti-hsa-miR146a or anti-hsa-miR378. Only transcripts which were predicted *in silico* to be targets of the selected miRNAs are presented. The y-axis represents the fold-change, relative to the corresponding control-miRNA (control pre-miR for miR129-5p and control anti-miR for miR146a and 378).
(TIF)

Table S1 **Effects of selected miRNAs on transcript levels of target genes in THP-1 cells, illustrated by fold changes in response to transfection with pre-hsa-miR129, anti-hsa-miR146a and anti-hsa-miR378.** THP-1 cells were stimulated with the corresponding stimulus to reflect the initial result in primary cells (MDP for cells transfected with pre-hsa-miR129-5p; TNF-α for cells transfected with anti-has-miR146a and anti-has-miR378).
(DOC)

Table S2 ***In silico*** **predictions of target genes for hsa miR-129-5p, 146a and 378.** The total counts of the predictions by 5 different published algorithms are listed. The algorithms used are: miRanda (Type: Complementary; John et al., 2004) miRanda miRBase (Type: Complementary; Enright et al., 2003); Target Scan (Type: Seed Complementary; Lewis et al., 2005); PicTar (Type: Thermodynamics; Krek et al., 2005); PITA (Type: Thermodynamics, 2ndary structure; Kertesz et al, 2007). For all the genes presented a transcript quantification after transfection with the corresponding pre or anti-miRs was performed.
(DOC)
