# Supplementary figures and images for: Microbial Pattern Recognition Causes Distinct Functional Micro-RNA Signatures in Primary Human Monocytes
Source: PLoS One. 2012 Feb 17;7(2):e31151. doi: 10.1371/journal.pone.0031151 (PMC3281918; doi:10.1371/journal.pone.0031151)

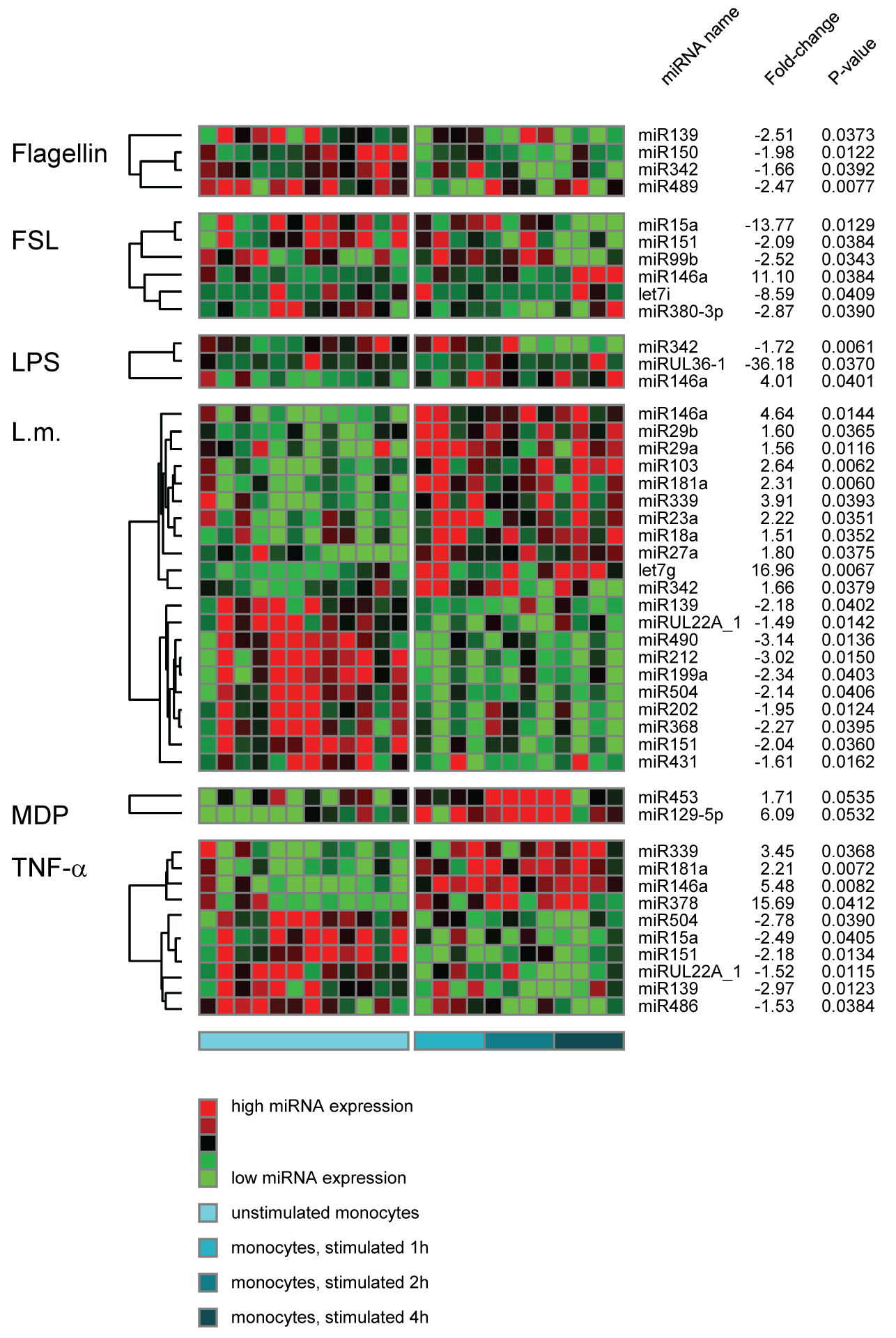

Supplement: Figure S1 — Response clusters illustrating all miRNAs responding significantly to a specific stimulus in primary monocytes. Flagellin, FSL (diacylated lipopeptide FSL-1), LPS (lipopolysaccharide), L.m. (Listeria monocytogenes), MDP (muramyl dipeptide), TNF-α (tumor necrosis factor alpha). Micro-RNA expression (arranged in rows) and for each sample (arranged in columns) are colored according expression intensity: red (high expression), green (low expression). To better visualize the expression differences within one miRNA, colors were based on normalized expression intensity (z-score). Micro-RNA names, signed fold-changes of each gene (+/− representing up/down-regulation) and corrected p-values are listed in the right columns. The dendrogram (left) shows the similarity of the expression profile for each miRNA within its response cluster, based on the correlation. (TIF) [file pone.0031151.s001.tif]

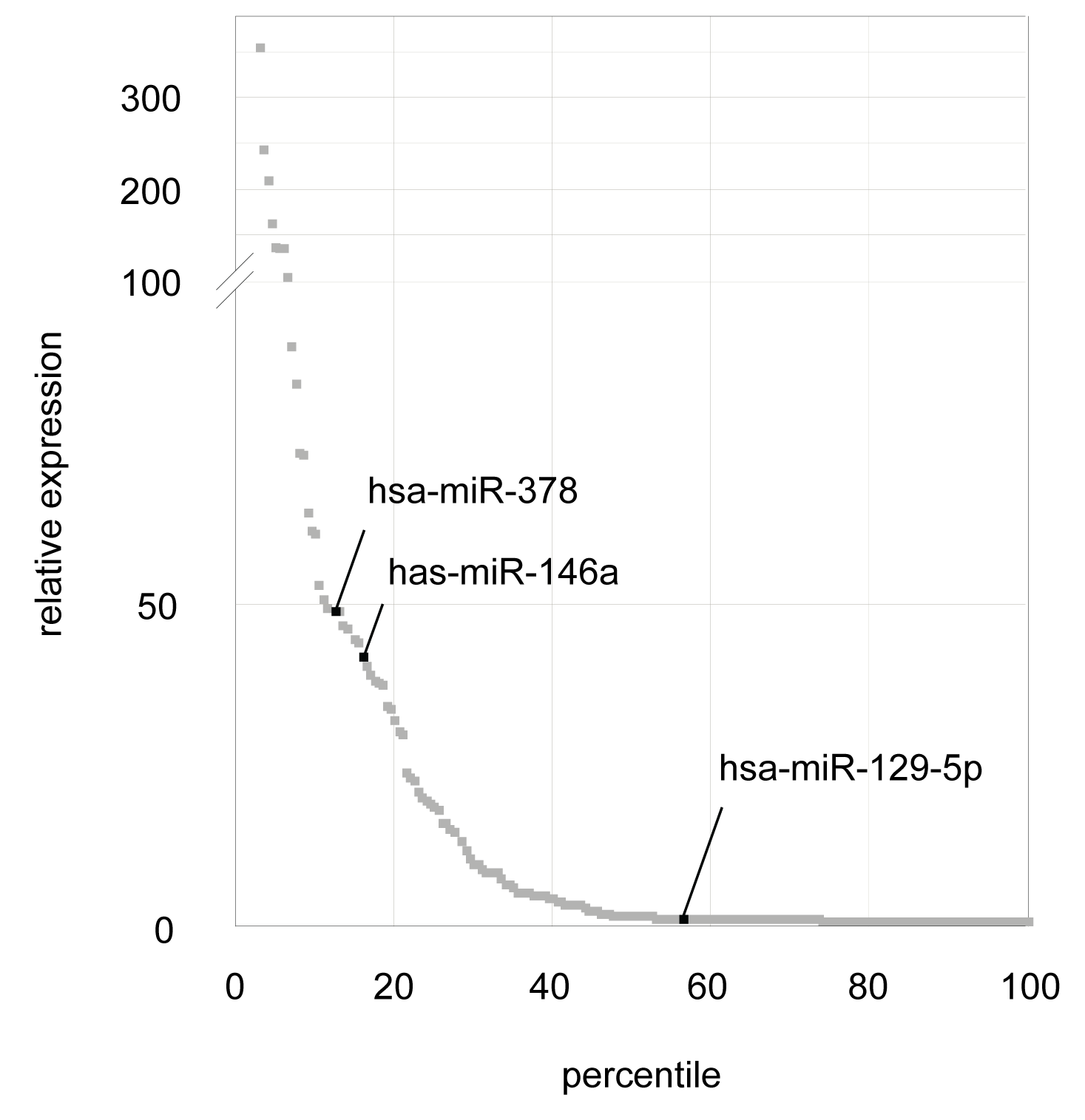

Supplement: Figure S2 — Profile of expressed miRNAs in THP-1 cells, illustrating all 330 measured miRNAs and their relative rank position on a 1–100% scale. To display their relative endogenous expression, miR129-5p, miR-146a and miR-378 are highlighted. (TIF) [file pone.0031151.s002.tif]

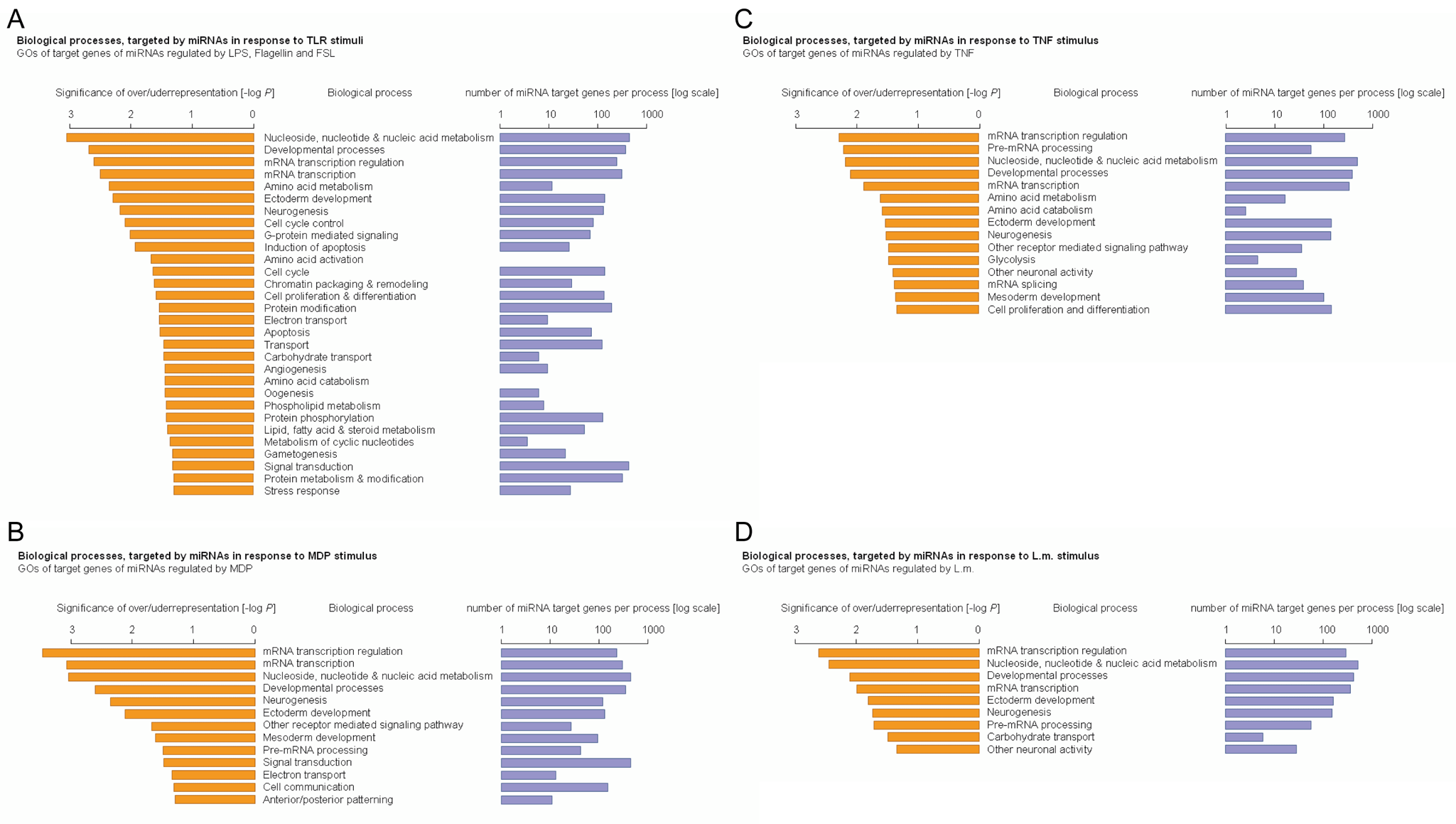

Supplement: Figure S3 — Biological processes of miRNA target genes. Results of a Gene Ontology analysis, where predicted target genes of each miRNA were associated to biological processes. The significance of the enrichment or the depletion of a biological process is displayed as −log(p) (orange) and the number of genes observed in the process are displayed on the right (blue, log-scale). Gene Ontology analysis were seperated into 4 different miRNA response groups: Biological processes of target genes of miRNAs regulated by LPS, flagellin and FSL (A), regulated by MPD (B), regulated by TNF-α (C) and regulated by Listeria monocytogenes (D). (TIF) [file pone.0031151.s003.tif]

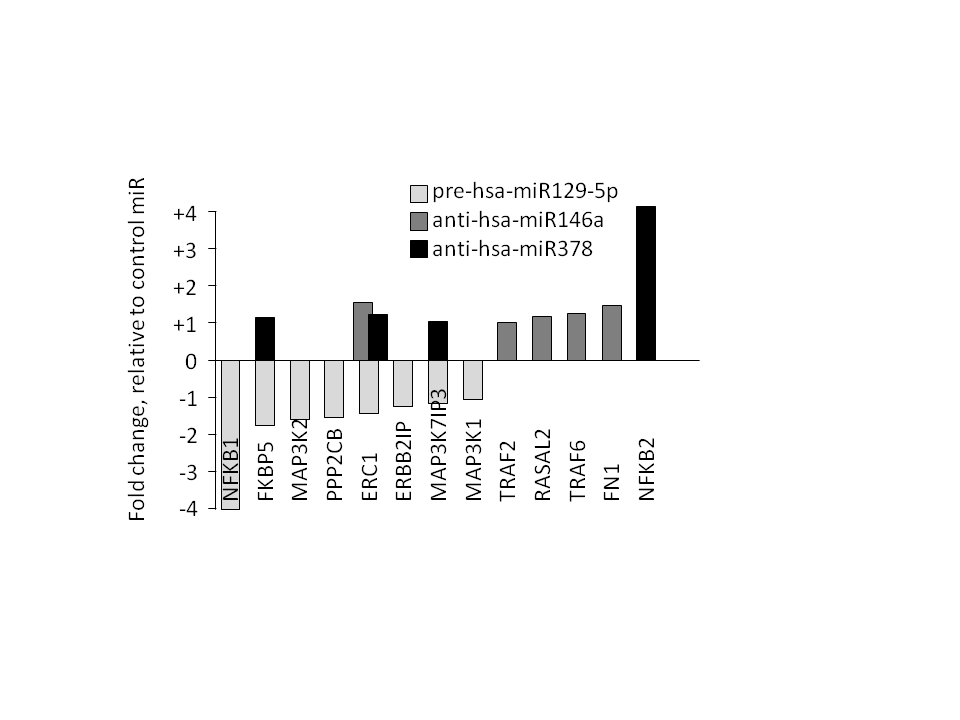

Supplement: Figure S4 — Quantitative responses of target genes in THP-1 cells. Responses were measured after transfecting cells with pre-hsa-miR129-5p, anti-hsa-miR146a or anti-hsa-miR378. Only transcripts which were predicted in silico to be targets of the selected miRNAs are presented. The y-axis represents the fold-change, relative to the corresponding control-miRNA (control pre-miR for miR129-5p and control anti-miR for miR146a and 378). (TIF) [file pone.0031151.s004.tif]
