# Supplementary material for: Microbial Pattern Recognition Causes Distinct Functional Micro-RNA Signatures in Primary Human Monocytes
Source: PLoS One. 2012 Feb 17;7(2):e31151. doi: 10.1371/journal.pone.0031151 (PMC3281918; doi:10.1371/journal.pone.0031151)
Supplement: Table S1 — Effects of selected miRNAs on transcript levels of target genes in THP-1 cells, illustrated by fold changes in response to transfection with pre-hsa-miR129, anti-hsa-miR146a and anti-hsa-miR378. THP-1 cells were stimulated with the corresponding stimulus to reflect the initial result in primary cells (MDP for cells transfected with pre-hsa-miR129-5p; TNF-α for cells transfected with anti-has-miR146a and anti-has-miR378). (DOC) [file pone.0031151.s005.doc]

**Supplemental Table 1:** Effects of selected miRNAs on transcript levels of target genes in THP-1 cells, illustrated by fold changes in response to transfection with pre-hsa-miR129, anti-hsa-miR146a and anti-hsa-miR378. THP-1 cells were stimulated with the corresponding stimulus to reflect the initial result in primary cells (MDP for cells transfected with pre-hsa-miR129-5p; TNF-α for cells transfected with anti-has-miR146a and anti-has-miR378).

| **GeneSymbol** | **pre-miR129-5p** | **anti-miR146a** | **anti-miR378** |
| --- | --- | --- | --- |
| BSG | 1.32 | -1.47 | -1.64 |
| BTRC | -14.75 | 1.11 | -1.22 |
| CAMP | 5.05 | -1.49 | -7.37 |
| CARD12 | 1.19 | -1.7 | -1.01 |
| CARD15 | -3.23 | 3.75 | 3.04 |
| CARD4 | -1.39 | -1.61 | -1.69 |
| CARD8 | -1.04 | -1.21 | -1.44 |
| CARD9 | -1.31 | -1.52 | -2.37 |
| CD14 | -2.32 | 1.79 | 1.74 |
| CD36 | -2.38 | 1.51 | 1.31 |
| CDC37 | -1.3 | -1.35 | -1.75 |
| CENTB1 | -29.28 | -2.07 | -3.13 |
| CHUK | 1.2 | -2.53 | -3.34 |
| CUL1 | -2.46 | -1.54 | -2.28 |
| DEFA5 | - | - | - |
| DEFB1 | 3.43 | -5 | -5.92 |
| DEFB103B;DEFB103A | - | - | - |
| DEFB4 | - | - | - |
| DUOX2 | -337.44 | 2.03 | 1.06 |
| DUSP16 | -1.47 | 1.56 | 1.06 |
| ERBB2IP | -1.24* | 1.31 | 1.09 |
| ERC1 | -1.43* | 1.58* | 1.25* |
| F2RL1 | - | - | - |
| FBXW7 | 39.12 | -1.49 | -1.93 |
| FKBP5 | -1.77* | 1.93 | 1.17* |
| FN1 | 1.22 | 1.49* | 1.52 |
| HERC4 | -9.12 | -1.26 | -1.76 |
| HSP90AA1 | -1.05 | -2.26 | -2.67 |
| HSP90AB1 | 1.05 | -1.1 | -1.4 |
| IER3 | -28.17 | 1.9 | 2.44 |
| IFNG | - | - | - |
| IKBKB | -1.19 | -1.38 | -1.69 |
| IKBKE | -3.77 | 2.45 | 2.49 |
| IKBKG | 2.58 | -1.03 | -1.39 |
| IL1B | -3.18 | 3.21 | 3.44 |
| IL32 | -224.08 | 182.49 | 149.17 |
| IL4 | - | - | - |
| IL8 | -8.06 | 2.2 | 2.78 |
| INPP4A | -1.79 | -1.18 | -1.4 |
| IQGAP2 | -1.21 | 1.32 | -1.13 |
| IRAK1 | 182.3 | -2.78 | -3 |
| IRAK4 | -1.68 | -1.51 | -1.7 |
| IRF7 | -1.91 | -7.33 | -43.8 |
| LYZ | 1.48 | -2.12 | -2.42 |
| MAP3K1 | -1.05* | -1.49 | -1.66 |
| MAP3K14 | -30.59 | 1.63 | -1.38 |
| MAP3K2 | -1.61* | -1.15 | -1.33 |
| MAP3K3 | -2.3 | 1.15 | -1.19 |
| MAP3K5 | -1.86 | -1.06 | -1.16 |
| MAP3K7 | - | -1.31 | -1.73 |
| MAP3K7IP1 | -1.57 | -1.79 | -2.44 |
| MAP3K7IP2 | -1.09 | -1.86 | -1.66 |
| MAP3K7IP3 | -1.17* | 1.18 | 1.06* |
| MAP3K8 | 1.49 | -1 | -1.54 |
| MCM5 | -1.27 | -1.58 | -1.58 |
| MCM7 | -1.46 | -1 | -1.27 |
| MEFV | -2.1 | 1.36 | 1.1 |
| MYD88 | - | 2 | 1.79 |
| NDUFA13 | -1.02 | -1.95 | -2.32 |
| NFKB1 | -4.05* | 2.67 | 3 |
| NFKB2 | 1.68 | 6.24 | 4.16* |
| NFKBIA | -5.45 | 3.34 | 3.07 |
| NFKBIB | -3.49 | 1.03 | -1.52 |
| NFKBIE | -4.52 | 2.8 | 2.84 |
| NLRC3 | -1.53 | -1.17 | -2.12 |
| NLRC4 | 1.39 | -1.57 | 1.1 |
| NLRC5 | -3.02 | -1.02 | -1.02 |
| NLRP1 | -2.15 | -2.35 | -2.59 |
| NLRP3 | -1.06 | -1.33 | -1.33 |
| NOD1 | 1.19 | -1.35 | -1.58 |
| NOD2 | -3.08 | 3.86 | 3.49 |
| NOX1 | - | - | - |
| PGLYRP1 | - | - | - |
| PGLYRP2 | - | - | - |
| PPM1D | -1.03 | -1.49 | -1.68 |
| PPM1J | - | -1.46 | - |
| PPM1L | -1.31 | -1.26 | -1.35 |
| PPP1R12C | -1.16 | -1.63 | -1.17 |
| PPP1R7 | -1.37 | -1.1 | -1.6 |
| PPP2CA | 9.59 | -1.69 | -1.93 |
| PPP2CB | -1.55* | -1.83 | -2.03 |
| PPP2R1A | -1.36 | 1 | -1.33 |
| PPP2R1B | -1.16 | -1.28 | -1.37 |
| PPP2R2D | -1.45 | -1.31 | -1.58 |
| PPP2R5C | 1.47 | -1.64 | -1.96 |
| PPP4C | -1.21 | -1.55 | -1.47 |
| PPP6C | 1.02 | 1.03 | -1.01 |
| PRDX4 | 1.2 | -3.48 | -3.88 |
| PTEN | -1.08 | -95.9 | -314.47 |
| PTPN2 | - | - | - |
| PTPRJ | -1.2 | 1.05 | -1.42 |
| PTPRN | - | - | - |
| RAC1 | -1.3 | -1.15 | -2.1 |
| RASAL2 | -1.15 | 1.2* | -1.13 |
| REL | -2.2 | -1.63 | -5.77 |
| RELA | -1.06 | 1.6 | 1.4 |
| RELB | -8.34 | 4.79 | 3.2 |
| RIPK1 | -2.05 | 1.17 | -1.13 |
| RIPK2 | -1.28 | -1.3 | -1.47 |
| RIPK3 | 1.45 | -1.36 | -1.4 |
| RNASE7 | - | - | - |
| RPL30 | 10.11 | -1.39 | -1.49 |
| S100A7 | - | - | - |
| SFTPA1B | - | 1.48 | - |
| SKP1A | - | - | - |
| SPAG9 | -1.2 | -1.31 | -1.68 |
| SUGT1 | 1.43 | -1.75 | -1.8 |
| TANK | -2.12 | -1.39 | -1.47 |
| TBK1 | -1.76 | -1.06 | -1.82 |
| TBKBP1 | -1.15 | -3.31 | -17.67 |
| TGFB1 | -1.57 | 1.17 | 1.09 |
| TICAM1 | -3.29 | -1.33 | -1.78 |
| TIRAP | -1.1 | -1.23 | -1.58 |
| TLR1 | -2.04 | -1.11 | 1.05 |
| TLR2 | -1.82 | 1.58 | 1.09 |
| TLR3 | - | - | - |
| TLR4 | 1.27 | -1.83 | -1.78 |
| TLR6 | -1.29 | -1.36 | -1.43 |
| TLR7 | -4.33 | 2.02 | 2.55 |
| TLR9 | 1.48 | -1.72 | -1.31 |
| TNF | -2.91 | 1.61 | 1.82 |
| TNFAIP3 | -11.3 | 9.2 | 7.48 |
| TNFRSF1A | -1.74 | 1.2 | 1.21 |
| TNIP2 | -1.31 | -1.25 | -1.71 |
| TOLLIP | 1.12 | -1.78 | -2.03 |
| TRAF1 | - | 4.62 | 1.47 |
| TRAF2 | -1.42 | 1.04* | -1.68 |
| TRAF3 | -2.16 | 1.16 | 1.11 |
| TRAF6 | -7.54 | 1.28* | -2.12 |
| TRAF7 | 1.52 | 1.16 | -1.05 |
| USP2 | 1.51 | 1.15 | -1.16 |
| ZNF141 | -1.58 | 1.12 | -1.33 |

Presented signed fold changes are based on ratios (stimulated vs. control), a star (*) indicates that this transcript was predicted by the target of the corresponding miRNA and that the fold change trend is in concordance with the prediction, a dash (-) indicates that no regulation was observed for this transcript.
