# Supplementary material for: Microbial Pattern Recognition Causes Distinct Functional Micro-RNA Signatures in Primary Human Monocytes
Source: PLoS One. 2012 Feb 17;7(2):e31151. doi: 10.1371/journal.pone.0031151 (PMC3281918; doi:10.1371/journal.pone.0031151)
Supplement: Table S2 — In silico predictions of target genes for hsa miR-129-5p, 146a and 378. The total counts of the predictions by 5 different published algorithms are listed. The algorithms used are: miRanda (Type: Complementary; John et al., 2004) miRanda miRBase (Type: Complementary; Enright et al., 2003); Target Scan (Type: Seed Complementary; Lewis et al., 2005); PicTar (Type: Thermodynamics; Krek et al., 2005); PITA (Type: Thermodynamics, 2ndary structure; Kertesz et al, 2007). For all the genes presented a transcript quantification after transfection with the corresponding pre or anti-miRs was performed. (DOC) [file pone.0031151.s006.doc]

| **Gene Symbol** | **Gene**  **Name** | **Number of *in silo* prediction to be a target of hsa miR-** | | |
| --- | --- | --- | --- | --- |
|  |  | **129-5p** | **146a** | **378** |
|  |  |  |  |  |
| CHUK | conserved helix-loop-helix ubiquitous kinase | 1 | 0 | 0 |
| ERBB2IP | erbb2 interacting protein | 1 | 0 | 0 |
| ERC1 | ELKS/RAB6-interacting/CAST family member 1 | 1 | 1 | 1 |
| FBXW7 | F-box and WD repeat domain containing 7 | 1 | 0 | 0 |
| FKBP5 | FK506 binding protein 5 | 1 | 0 | 1 |
| FN1 | fibronectin 1 | 0 | 1 | 0 |
| IRAK1 | interleukin-1 receptor-associated kinase 1 | 0 | 3 | 0 |
| LYZ | lysozyme (renal amyloidosis) | 0 | 1 | 0 |
| MAP3K1 | mitogen-activated protein kinase kinase kinase 1 | 1 | 0 | 0 |
| MAP3K2 | mitogen-activated protein kinase kinase kinase 2 | 1 | 0 | 0 |
| MAP3K7 | mitogen-activated protein kinase kinase kinase 7 | 1 | 0 | 0 |
| MAP3K7IP3 | mitogen-activated protein kinase kinase kinase 7 (…) | 1 | 0 | 1 |
| MAP3K8 | mitogen-activated protein kinase kinase kinase 8 | 0 | 1 | 0 |
| NFKB1 | nuclear factor of kappa light polypeptide gene (…) | 1 | 0 | 0 |
| NFKB2 | nuclear factor of kappa light polypeptide gene (…) | 0 | 0 | 1 |
| PPM1D | protein phosphatase 1D magnesium-dependent (…) | 0 | 1 | 0 |
| PPP1R7 | protein phosphatase 1, regulatory (inhibitor) subunit 7 | 0 | 1 | 0 |
| PPP2CB | protein phosphatase 2 (formerly 2A), catalytic (…) | 1 | 0 | 0 |
| PPP2R2D | protein phosphatase 2, regulatory subunit B, (…) | 0 | 0 | 1 |
| PRDX4 | peroxiredoxin 4 | 0 | 1 | 0 |
| PTPN2 | protein tyrosine phosphatase, non-receptor type 2 | 0 | 1 | 0 |
| RASAL2 | RAS protein activator like 2 | 0 | 2 | 0 |
| RPL30 | ribosomal protein L30 | 1 | 0 | 0 |
| SKP1A | S-phase kinase-associated protein 1 | 1 | 0 | 0 |
| TLR3 | toll-like receptor 3 | 0 | 1 | 0 |
| TRAF2 | TNF receptor-associated factor 2 | 0 | 1 | 0 |
| TRAF6 | TNF receptor-associated factor 6 | 0 | 3 | 1 |

Supplementary table 1: *In silico* predictions of target genes for hsa miR-129-5p, 146a and 378. The total counts of the predictions by 5 different published algorithms are listed. The algorithms used are: miRanda (Type: Complementary; John et al., 2004) miRanda miRBase (Type: Complementary; Enright et al., 2003); Target Scan (Type: Seed Complementary; Lewis et al., 2005); PicTar (Type: Thermodynamics; Krek et al., 2005); PITA (Type: Thermodynamics, 2ndary structure; Kertesz et al, 2007). For all the genes presented a transcript quantification after transfection with the corresponding pre or anti-miRs was performed.
